# Evaluation of qPCR reference genes for taimen (*Hucho taimen*) under heat stress

**DOI:** 10.1038/s41598-021-03872-x

**Published:** 2022-01-10

**Authors:** Xiaoxing Yang, Guangxiang Tong, Le Dong, Ting Yan, Huan Xu, Guopan Tang, Yongquan Zhang, Kai Ma, Jiasheng Yin, Youyi Kuang

**Affiliations:** 1 Heilongjiang River Fisheries Research Institute, Chinese Academy of Fishery Sciences, Harbin, 150070 China; 2 Key Laboratory of Freshwater Aquatic Biotechnology and Breeding, Ministry of Agriculture and Rural Affairs, Harbin, 150070 China; 3 Key Open Laboratory of Cold Water Fish Germplasm Resources and Breeding of Heilongjiang Province, Harbin, 150070 China; 4 Henan University of Animal Husbandry and Economy, Zhengzhou, 450016 China; 5 College of Fisheries and Life Science, Shanghai Ocean University, Shanghai, 201303 China

**Keywords:** Genetics, Molecular biology

## Abstract

As a powerful and attractive method for detecting gene expression, qRT-PCR has been broadly used in aquaculture research. Understanding the biology of taimen (*Hucho taimen*) has drawn increasing interest because of its ecological and economic value. Stable reference genes are required for the reliable quantification of gene expression, but such genes have not yet been optimized for taimen. In this study, the stability levels of 10 commonly used candidate reference genes were evaluated using geNorm, NormFinder, BestKeeper, and RefFinder. The expression levels of the 10 genes were detected using 240 samples from 48 experimental groups consisting of 40 individuals treated under four heat-stress conditions (18, 20, 22, and 24 °C) for 24 h and 26 °C for 4, 24, 48, and 72 h. Six tissues (blood, heart, brain, gill, skin, and liver) were collected from each individual. Ribosomal protein S29 (*RPS29*) and ribosomal protein L19 (*RPL19*) were the most stable genes among all of the samples, whereas 28S ribosomal RNA (*28S rRNA*), attachment region binding protein (*ARBP*), and 18S ribosomal RNA (*18S rRNA*) were the least stable. These results were verified by an expression analysis of taimen heat-stress genes (heat shock protein 60, *hsp60*, and heat shock protein 70, *hsp70*). In conclusion, *RPS29* and *RPL19* are the optimal reference genes for qRT-PCR analyses of taimen, irrespective of the tissue and experimental conditions. These results allow the reliable study of gene expression in taimen.

## Introduction

Taimen (*Hucho taimen*), belonging to Salmonidae, is a cold freshwater carnivorous fish^1^. In recent decades, the wild taimen population has decreased drastically due to over-exploitation and environmental pollution^2^. Taimen has been classified as a vulnerable species in the "China Red Book of Endangered Animals" and "China's Red List of Species."^3^ Taimen is also an economically important fish that has been extensively cultured in China since 2003 because of its fast growth, nutritional value, and good meat taste and quality^4^. Water temperature has been found to be an important factor that influences taimen growth and survival, and the optimal water temperature for taimen growth is 15–18 °C. When the water temperature exceeds 18 °C, taimen refuse to feed, and the growth rate decreases as the temperature increases. The fish may even die from high temperature stress^5^. Therefore, it is important to study the high-temperature tolerance mechanisms of taimen to optimize the production of this species, including breeding programs. Currently, many microsatellite^6,7^ markers, transcriptomes obtained by mRNA sequencing^8^, high resolution linage maps^9^, and genome sequences of huchen, a closely related fish (*Hucho hucho*, GCA_003317085.1 in GenBank) have been developed. These are useful tools to characterize candidate genes related to high-temperature tolerance. Although there are many methods to study the molecular mechanism of high-temperature tolerance in a species, quantitative real-time PCR (qRT-PCR) is an essential tool to investigate the role of a gene in high-temperature tolerance^10^.

qRT-PCR is a nucleic acid quantification technology developed in accordance with traditional PCR technology^11^. It is characterized by high sensitivity, good repeatability, and specificity; in addition, it has high-throughput capabilities and has been broadly applied to gene expression analyses and clinical diagnoses^12^. The accuracy and reliability of qRT-PCR are often dependent on many factors^13^, including RNA quality, reverse transcription efficiency, and appropriate reference gene selection. In a gene expression analysis, the selection of an appropriate reference gene is a crucial prerequisite for accurately quantifying expression levels using qRT-PCR^14^. There are many reference genes used in gene expression analysis. Genes involved in the cytoskeleton and fundamental biological processes are frequently used as reference genes, such as *18S rRNA*, *28S rRNA*, *actins*, glyceraldehyde-3-phosphate dehydrogenase (*GAPDH*)^15,16^, *α-tubulin*^17^, and *ARBP*^18^. Filby et al.^19^ recommend the use of *18S rRNA*, ribosomal protein l8 (*rpl8)*, hypoxanthine phosphoribosyltransferase 1 (*hprtI),* and tata box binding protein (*tbp)* as the reference genes in qRT-PCR studies of the effects of estrogen in fish. Ma^20^ et.al thought beta-actin (*β-actin*) and elongation factor EF1 alpha (*ef1-α*) were the best reference genes for qRT-PCR analysis in the liver and head kidney of rainbow trout (*Oncorhynchus mykiss*) under heat stress. Liao et al.^21^. believed that *RPL19*, *ef1-α*, *18S rRNA*, and *RPL13* were suitable reference genes for the study of tiger puffer (*Takifugu rubripes*) in different tissues. Pei et al.^22^ reported that *GAPDH* could be used as a suitable reference for nuclear reprogramming in zebrafish (*Danio rerio*).

Generally, the optimal reference genes should be stably expressed in all of the organs under the various test conditions. However, a growing number of research reports have suggested that the stability levels of traditional housekeeping genes, which have served as reference genes, are inconsistent under different experimental conditions. For example, Olsvik et al.^23^ evaluated six reference genes, including *18S*, S20 ribosomal protein (*S20*), *β-actin*, *GAPDH*, and two paralog genes encoding elongation factor 1A (*EF1A*_*A*_ and *EF1A*_*B*_), in eight tissues (gill, liver, head kidney, spleen, thymus, brain, muscle, and posterior intestine) of Atlantic salmon (*Salmo salar*) cultured in normal or smoltification conditions, and they found that *EF1A*_*B*_ was the best reference gene, whereas Jorgensen et al.^24^ reported that the combination of *18S rRNA*, *EF1A*, and RNA polymerase I (*RPL1*) was the best normalization method for qRT-PCR in immune-related organs despite viral infection. Ma et al.^20^ and Shekh et al.^25^ also demonstrated that the optimum reference genes for rainbow trout varied in different tissues and experimental conditions. Therefore, it is essential to characterize and evaluate whether selected candidate reference genes are stably expressed under different test conditions and yield reliable results^26^.

Here, initially, the practical problem of using qRT-PCR technology for taimen research was addressed. We used four methods, geNorm^27^, NormFinder^28^, BestKeeper^29^, and RefFinder^30^, to evaluate the stability of 10 candidate reference genes in different taimen tissues under different temperature conditions. To validate the selected reference genes, the heat shock protein genes^31^ *hsp60* and *hsp70* were selected as targets to assess the performance of the selected reference genes in different conditions.

## Methods and materials

### Ethics statement

All of the experiments involving the handling and treatment of fish in this study were approved by the Animal Care and Use Committee of the Heilongjiang River Fisheries Research Institute of Chinese Academy of Fishery Sciences (HRFRI). The methods were carried out in accordance with approved guidelines. Before samples were collected, all of the fishes were euthanized in fresh 250 mg/L MS222 solution (Sigma, Darmstadt, Germany). In addition, we followed the ARRIVE guidelines (https://arriveguidelines.org).

### Fish source and heat-stress experimental settings

**Figure 1:**
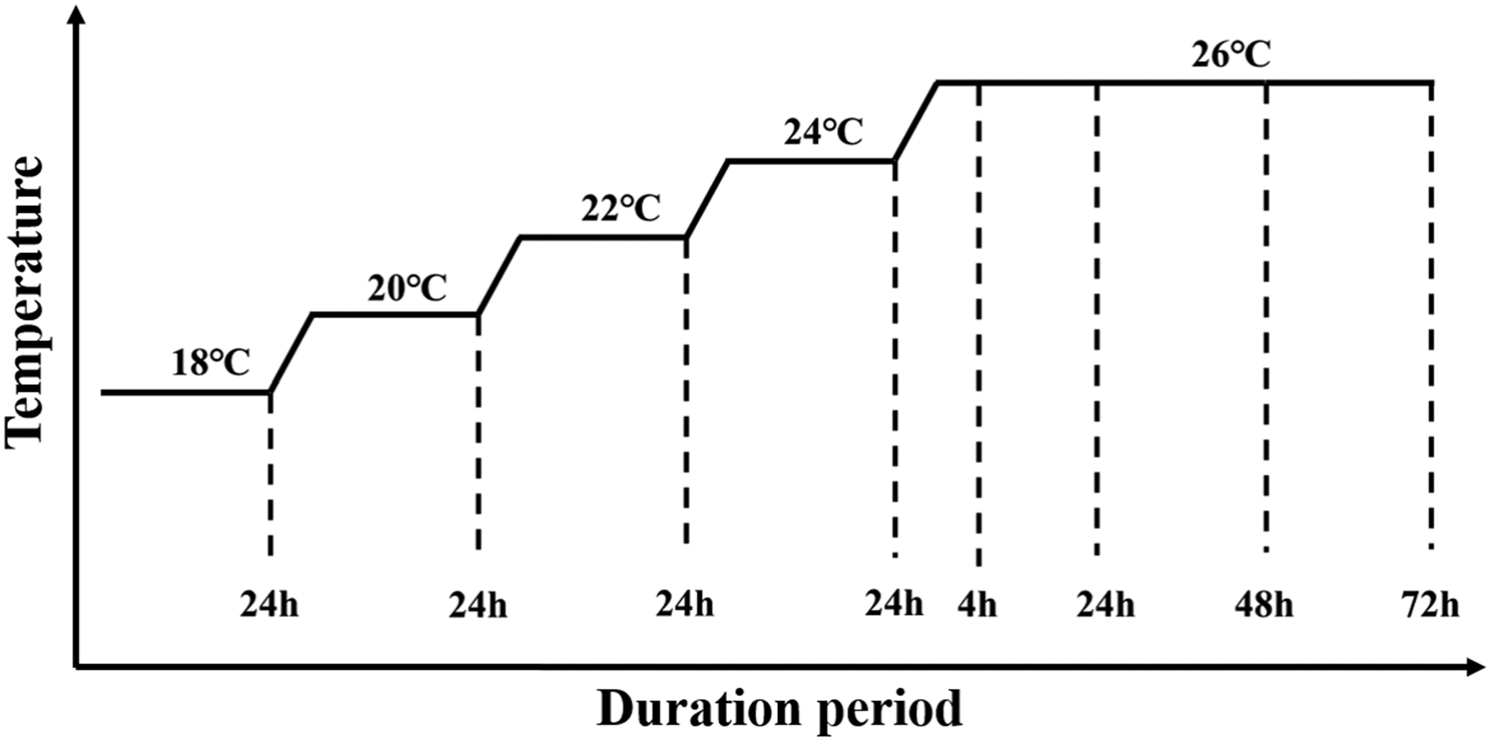
Heat stress experimental procedure. The X-axis represents the duration of the temperature stress, the Y-axis represents the temperature, and the dashed lines indicate the sampling time points.

The fishes used in this study were cultured at the Bohai Cold Fish Experimental Station of the Heilongjiang River Fisheries Research Institute of Chinese Academy of Fishery Sciences(HRFRI). Sixty healthy individuals with body weights of 15 ± 5.0 g were used to carry out high-temperature stress experiments. The fishes were first cultured for 3 weeks at 18 °C in a recirculating culture system containing three 0.3 m^3^ tanks to acclimate to the environment. They were fed a commercial diet twice a day throughout the study. For temperature-stress experiments, 60 individuals were randomly divided into 8 groups. Heat stress began in all eight groups simultaneously. Fishes of each group were also cultured in a 0.3 m^3^ tank with recirculation, water filtration, an oxygen supplier, and temperature control. During the experiment, the dissolved oxygen was maintained at higher than 8 mg/L, and the ammonia concentration was maintained below 0.01 mg/L by exchanging water with the same temperature. Five temperatures in a 2 °C gradient, 18, 20, 22, 24, and 26 °C, were used, and the water temperature was controlled at ± 0.5 °C with an automatic water temperature controller (Sensen Group Co., Ltd., China). Fishes were randomly sampled after 24 h in water at the target temperatures of 18, 20, 22, and 24 °C. Our pilot study found that the fishes started to die when the water temperature rose to 26 °C. Thus, we took samples at 4, 24, 48, and 72 h as the water temperature rose from 24 to 26 °C. In summary, a total of eight heat-stress experiments (named temperature–time) were carried out (Fig. 1): 18 °C-24 h, 20 °C-24 h, 22 °C-24 h, 24 °C-24 h, 26 °C-4 h, 26 °C-24 h, 26 °C-48 h, and 26 °C-72 h. In each experiment, five fishes were randomly chosen for tissue dissection.

### Sample collection and RNA extraction

For each individual, six tissues, namely, the liver, blood, heart, brain, gill, and skin were collected for total RNA extraction. After euthanizing the fish with 250 mg/L fresh MS222 (Sigma, Darmstadt, Germany) solution, the peripheral blood was first collected from the caudal vein using a syringe and was mixed with 1:1 anticoagulant (30 mM disodium citrate, 0.34 M NaCl, and 10 mm EDTA-Na_2_; pH 7.5). The other five tissues were then dissected. The tissues were stored in liquid nitrogen until total RNA was extracted using TRIzol reagent (Thermo Fisher, CA, USA) following the recommended protocol^17^. After treatment with DNaseI (Fermentas, MD, USA) to eliminate genomic DNA, the RNA quality was assessed using a 1.0% agarose gel and a Nano Drop 8000 Spectrophotometer (Thermo Fisher, CA, USA), and the RNA quantity was measured using a Qubit3 kit (Thermo Fisher, CA, USA). The RNA with an OD260/OD280 value that ranged from 1.8 to 2.0, and a concentration ≥ 200 ng/μL was considered for qRT-PCR analysis.

### Reference gene selection and primer design

Using the transcriptome published by Tong^8^, the transcript sequences of 10 genes, *28S rRNA*, *GAPDH*, *ARBPR*, *18S rRNA*, *β*-*actin*, *RPS29*, *RPL13*, *RPL19*, *Saha*, and *α-tubulin*, were determined for qRT-PCR primer design. The primers were first designed using Primer3 https://primer3plus.com/ (Table 1) and then aligned to the taimen transcriptome using a BLAST search to confirm the specificity^32^. Primers that matched non-target genes with less than three mismatched bases were eliminated. The primers were synthesized by GENEWIZ Co. (Suzhou, China).

**Table 1:** Primer sequences and qPCR efficiencies of the 10 candidate taimen reference genes.

Accession number	Gene (Full Name)	Lift primer (5′-3′)	Right primer (5′-3′)
HAGJ01000001	*18S rRNA* (18S ribosomal RNA)	CGTTCTTAGTTGGTGGAGCG	AACGCCACTTGTCCCTCTAA
HAGJ01162991	*Saha* (S-adenosyl-L-homocysteine hydrolase)	TGGAGGGATGGCTGAACATT	AGAGCACTGGAGGAAACACA
HAGJ01029436	*GAPDH* (Glyceraldehyde-3-phosphate dehydrogenase)	GTCTTCTGGGTAGCGGTGTA	ACCATCGTCAGCAATGCATC
HAGJ01015081	*RPL13* (ribosomal protein L13)	GGCCATCTTGAGTTCCTCCT	GCACCATTGGCATCTCTGTT
HAGJ01017568	*ARBP* (attachment region binding protein)	GGGCTTTGTCTTCACCAAGG	CTTCTCAGGACCAAGCCCAG
HAGJ01147709	*28S RNA* (28S ribosomal RNA)	GTCCTTCTGATCGAGGCTCA	GGAGTTTACCACCCGCTTTG
HAGJ01168534	*β-actin* (actin, beta)	TCTACGAAGGCTACGCTCTG	CAGCTTCTCCTTGATGTCGC
HAGJ01117629	*RPL19* (ribosomal protein L19)	ACACGGGCATAGGTAAGAGG	TCGATTTTCTTGGCCTCCCT
HAGJ01023957	*RPS29* (ribosomal protein S29)	TGGGACATCAGAGCCTCTAC	CTGGCGGCACATGTTGAG
HAGJ01091547	*α-tubulin* (alpha-Tubulin)	CGAGCCATACATCACACACG	TGCAATTGGGTGTTGATCCA
HAGJ01009947	*Hsp60* (heat shock protein 60)	GACATCATCAGACGAGCCCT	ACGTACTCTCCTTCCATGGC
HAGJ01160617	*Hsp70* (heat shock protein 60)	CCGCCTGGTTAGTCACTTTG	AGTGTTCTCTTGGCCCTCTC

#### Quantitative real-time PCR

The cDNA was synthesised using a RevertAid First-Strand cDNA Synthesis Kit (Fermentas, MD, USA) with oligo(dT)_18_ as the primer and stored at − 20 °C. Before the expression levels of the 10 candidate genes were detected using qRT-PCR, the specificity of the primers was confirmed using conventional PCR. The PCR was carried out in a 10 μL volume, which included 1 μL cDNA from the heart (50 ng/μL), 5 μL 2 × DreamTaq Green PCR master Mix (Thermo Fisher, CA, USA), 0.5 μL of each primer (10 µM), and 3 μL H_2_O. The amplification was carried out using an ABI9700 thermocycler (Thermo Fisher, CA, USA), and the PCR program was set as follows: 95 °C for 3 min, followed by 30 cycles of 95 °C for 30 s, 60 °C for 30 s, and 72 °C for 30 s, and a final temperature of 72 °C for 5 min. The PCR products were detected using 2% agarose gel electrophoresis.

The qRT-PCR was carried out in a 10-μL volume that included 1 μL cDNA (50 ng/μL), 5 μL 2 × Luna universal SYBR qPCR Master Mix (New England Biolabs, MA, USA), 0.5 μL of each primer (10 µM), and 3 μL H_2_O. The amplification program was as follows: 95 °C for 15 s, followed by 40 cycles of 95 °C for 15 s and 60 °C for 30 s. A melting curve was performed from 60 to 95 °C. The qRT-PCR was performed with a MicroAmp™ Optical 384-well Reaction Plate (Thermo Fisher, CA, USA) in QuantStudio Flex 6 (Thermo Fisher, CA, USA). Three technological replicates were used for each sample. To calculate the qRT-PCR efficiency of each gene, 10-, 100-, and 1,000-fold diluted cDNA samples were used to create a standard curve, and a linear regression model was built with the log10 (concentration) as the independent variable. The corresponding qRT-PCR efficiencies (*E*) were calculated using the following equation: *E* = [10(− 1/slope) − 1] × 100^33,34^.

#### Stability analysis of candidate reference genes

The relative expression levels of the candidate reference genes were calculated using Ct values and amplicon mean amplification efficiencies^24^. To assess the stability levels of the 10 reference genes, four programs, namely, geNorm^27^, NormFinder^28^, BestKeeper^29^, and RefFinder^30^, were used. The Ct values of the 10 candidate genes were analyzed from 48 experiments having 2 experimental factors. The first factor was the six tissues (blood, heart, brain, gill, skin, and liver), and the second factor was the eight sets of different heat-stress conditions: 18 °C-24 h, 20 °C-24 h, 22 °C-24 h, 24 °C-24 h, 26 °C-4 h, 26 °C-24 h, 26 °C-48 h, and 26 °C-72 h.

#### Expression of *hsp60* and *hsp70*

Heat shock proteins (HSPs) can decrease the oxidative stress induced by thermal stress^36^ and can be biomarkers of thermal stress^37^. Because peripheral blood mononuclear cells could be collected without harming the fish and expressed HSPs^38^, and HSPs play an important role in neurodevelopment and neuroinflammation^39–41^, which could protect the brain from heat stress, we analyzed the expressed levels of *hsp60* and *hsp70* in blood and brain tissues to validate the selected reference genes and verify whether *hsp60* and *hsp70* were related to heat stress in taimen and could be used as marker genes to study the physiological functions of taimen under heat stress.

We used samples from heat stress conditions of 18 °C-24 h, 20 °C-24 h, 22 °C-24 h, 24 °C-24 h, and 26 °C-24 h to perform the qRT-PCR of *hsp60* and *hsp70*, the samples from 18 °C-24 h were used as control. The qRT-PCR volume and program for *hsp60* and *hsp70* were the same as in the above description. The primers are listed in Table 1. The expression levels were calculated using 2^-ΔΔct^. To validate the selected reference genes, five different normalization methods were applied to blood and brain samples: normalization based on (i) the most stable gene; (ii) the second most stable gene; (iii) the first and second most stable genes; (iv) the most unstable gene; and (v) the second most unstable gene.

## Results and analysis

### Specificity of the candidate genes

**Figure 2:**
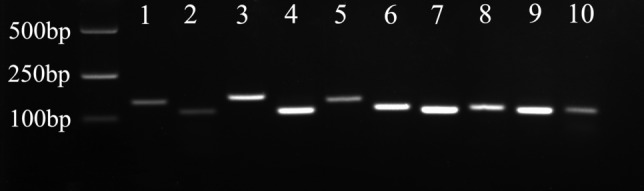
The specificities of the primer pairs for qRT-PCR amplification. The PCR product for each gene was confirmed using 2% agarose gel electrophoresis. Lane 1: *β-actin*; Lane 2: *RPL19*; Lane 3: *RPL13*; Lane 4: *RPS29*; Lane 5: *ARBP*; Lane 6: *α-tubulin*; Lane 7: *28S rRNA*; Lane 8: *GAPDH*; Lane 9: *18S rRNA*; Lane 10: *Saha.*

The specificity levels of the primers were confirmed using conventional PCR and 2% agarose gel electrophoresis. The results showed that a single band for each gene was detected, and no dimers or non-specific amplified bands occurred (Fig. 2), which indicated that the designed primers were appropriate for qRT-PCR.

### Quantitative real-time PCR analysis of reference genes

**Figure 3:**
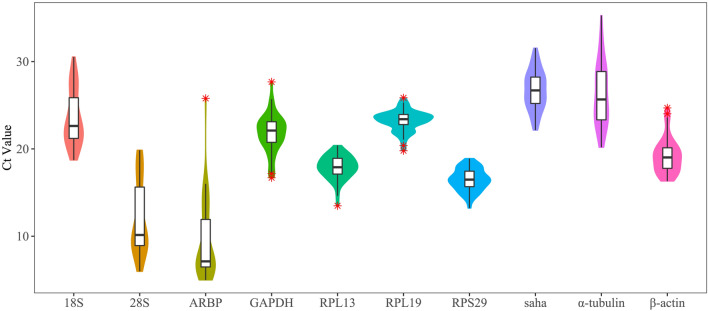
The distributions of the Ct values of 10 candidate reference genes in different experiments. The distributions are displayed using violin and box plots; * indicate the outliers.

The qRT-PCR analysis using the fluorescent SYBR dye showed that the melting curves of all of the amplicons presented single distinct signal peaks, which indicated that the primers for the 10 reference genes were appropriate for quantifying their expression levels. The expression abundance of each gene was represented by the Ct value, with a high Ct value indicating a low gene expression abundance^35^. The Ct values of the 10 candidate genes varied among tissues and heat-stress conditions, ranging from 4.99 to 30.53 (Fig. 3, Supplementary Figure S2 and S3). Transcripts of *ARBP* were the most abundant, followed by those of *28S rRNA* and *RPS29*, whereas those of *Saha* were the least abundant (Fig. 3, Supplementary Figure S2 and S3). In the skin, blood, and brain, *RPL13* presented the minimum coefficient of variation (CV) with mean CT values of 18.37, 19, and 16.84, respectively, and CV values of 0.77%, 0.75%, and 1.02%, respectively (Supplemental Table S1). In the heart, liver, and gill, *RPL19* presented the minimum CV with mean CT values of 22.78, 22.31, and 23.10, respectively, and CV values of 0.83%, 1.90%, and 0.66%, respectively (Supplemental Table S1). *RPS29* also presented the minimum CV (1.90%) in the liver with a mean CT value of 16.11 (Supplemental Table S1). Considering the different heat-stress conditions, *RPS29* was the gene with the minimum deviation of CT value under five experimental conditions, including 18 °C-24 h, 24 °C-24 h, 26 °C-4 h, 26 °C-24 h, and 26 °C-48 h, with mean CT values of 15.79, 17.08, 15.78, 15.65, and 18.02, respectively, and CV values of 1.08%, 0.81%, 0.51%, 1.61%, and 0.27%, respectively (Supplementary Table S2). To investigate whether the gene expression abundance was significantly different in tissues and under different heat stress conditions, we performed a two-way analysis of variance (ANOVA) for each gene and found that the CT values of each gene were significantly different in tissues and under different heat stress conditions. The statistical effect sizes of tissues ranged from 0.22 to 0.63, and those of heat stress condition ranged from 0.22 to 0.75 (Supplementary Table S3). A small effect size of a factor means the influence of this factor on CT values is negligible. According to the effect sizes of tissues and heat stress conditions, the expression abundance of α-tubulin was least affected by tissues, followed by 28S and RPL19, whereas the expression abundance of RPL19 was least affected by heat stress conditions, followed by GAPDH, RPL13 and RPS29. In total, *RPL19*, *RPS29*, and *RPL13* presented as more stable than the other genes despite the varying tissues and heat-stress conditions.

### Stability evaluation using geNorm

**Figure 4:**
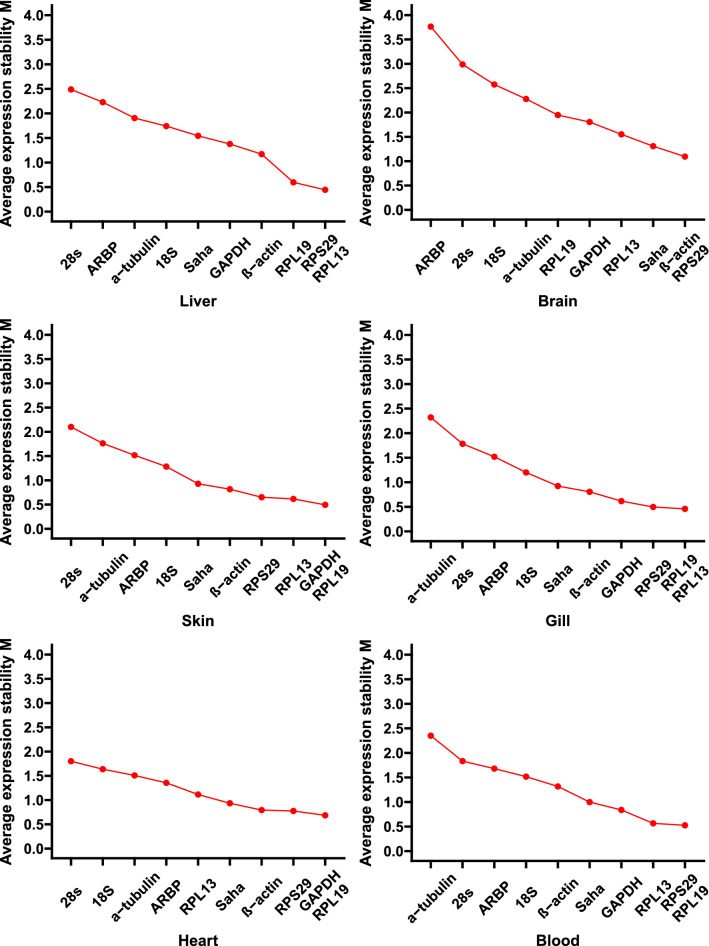
Expression stability values (M) of 10 candidate reference genes under heat-stress conditions as assessed by geNorm. Smaller M values represent more stable gene expression in different tissues.

The stability levels of the reference genes were assessed by computing the expression stability (*M*) values among samples using geNorm. A high *M* value represents less stability^27^. The *M* values calculated using geNorm software indicated that the most stable genes varied among the different experiments (Fig. 4). In the liver, *RPS29* and *RPL13* were the most stably expressed genes, whereas in the brain, *RPS29* and β-actin were the most stably expressed genes. In the skin and heart, *RPL19* and *GAPDH* were the most stably expressed genes. *RPL13* and *RPL19* were the most stably expressed genes in the gill, whereas *RSP29* and *RPL19* were the most stably expressed genes in the blood.

### Stability evaluation using NormFinder

The gene expression stability levels, as evaluated by NormFinder software, are listed in Table 2. The stability levels of genes differed among the 48 experiments. The most stable reference genes were *β-actin* and *RPS29* in the liver and brain, whereas the most unstable reference genes were *28S rRNA* and *ARBPR*. In the skin and heart, *RPS29* and *Saha* were the most stably expressed genes, whereas *28S RNA*, *18S rRNA*, and *α-tubulin* were the least stably expressed genes. In the gill and blood, *Saha* and *GAPDH* were the most stable reference genes, whereas *28s RNA*, *α-tubulin*, and *ARBP* were the most unstable reference genes.

**Table 2:** Expression stability values of 10 candidate taimen reference genes under heat-stress conditions as assessed by NormFinder.

Rank	Liver		Brain		Skin		Gill		Heart		Blood	
	Gene	Stable value	Gene	Stable value	Gene	Stable value	Gene	Stable value	Gene	Stable value	Gene	Stable value
1	*β-actin*	0.710	*β-actin*	0.379	*Saha*	0.115	*Saha*	0.309	*Saha*	0.208	*Saha*	0.234
2	*RPS29*	0.719	*RPS29*	0.641	*RPS29*	0.492	*GAPDH*	0.559	*RPS29*	0.319	*GAPDH*	0.252
3	*GAPDH*	0.909	*Saha*	0.993	*β-actin*	0.780	*RPS29*	0.651	*β-actin*	0.707	*β-actin*	0.680
4	*RPL13*	1.013	*α-tubulin*	1.537	*RPL13*	0.828	*18S*	0.740	*GAPDH*	0.786	*RPS29*	0.861
5	*18S*	1.061	*RPL13*	1.596	*GAPDH*	0.909	*β-actin*	0.940	*ARBP*	0.840	*18S*	0.982
6	*α-tubulin*	1.204	*GAPDH*	1.643	*18S*	0.963	*RPL19*	0.943	*RPL19*	0.894	*RPL19*	1.096
7	*RPL19*	1.233	*18S*	1.703	*RPL19*	1.052	*RPL13*	0.961	*α-tubulin*	1.061	*ARBP*	1.171
8	*Saha*	1.396	*RPL19*	1.831	*ARBP*	1.198	*ARBP*	1.332	*RPL13*	1.129	*RPL13*	1.258
9	*ARBP*	1.842	*28S*	2.694	*α-tubulin*	1.481	*28S*	1.745	*18S*	1.198	*28S*	1.542
10	*28S*	2.279	*ARBP*	4.506	*28S*	2.248	*α-tubulin*	2.944	*28S*	1.499	*α-tubulin*	2.913

### Stability evaluation using BestKeeper

The stable expression of the candidate reference genes was also analyzed using BestKeeper. In this program, the average Ct value of each reaction was used to analyze the stability of each candidate reference gene. The standard deviation (SD) and coefficient of variation (CV) were calculated by BestKeeper based on the Ct values^14^. The most stable reference genes were represented by the lowest CV and SD (CV ± SD) values. The gene ranking suggested by BestKeeper is shown in Table 3. In brief, *RPS29*, *RPL13*, and *RPL19* were determined to be the most reliable reference genes in all of the tissues under different heat-stress conditions, whereas *ARBP*, *28S rRNA*, *α-tubulin*, and *18S rRNA* showed the highest CV ± SD values, which suggested that their expression levels were very unstable.

**Table 3:** Expression stability values of 10 candidate reference genes calculated by the Best Keeper.

Rank	Liver		Brain		Skin		Gill		Heart		Blood	
	Gene	CV	Gene	CV	Gene	CV	Gene	CV	Gene	CV	Gene	CV
1	*RPL19*	1.54	*RPL13*	0.72	*RPL13*	0.58	*RPL19*	0.49	*RPL19*	0.59	*RPL19*	0.59
2	*RPL13*	1.61	*RPL19*	0.83	*RPL19*	0.67	*RPL13*	0.66	*GAPDH*	0.68	*RPL13*	0.60
3	*β-actin*	1.64	*RPS29*	1.09	*RPS29*	0.71	*RPS29*	0.68	*RPS29*	0.88	*RPS29*	0.68
4	*RPS29*	1.65	*β-actin*	1.39	*GAPDH*	0.84	*GAPDH*	0.84	*β-actin*	0.89	*GAPDH*	0.85
5	*GAPDH*	1.66	*GAPDH*	1.74	*β-actin*	0.98	*Saha*	1.06	*RPL13*	1.00	*Saha*	1.24
6	*Saha*	2.27	*Saha*	1.77	*Saha*	1.09	*β-actin*	1.23	*Saha*	1.23	*β-actin*	1.96
7	*α-tubulin*	2.38	*α-tubulin*	2.37	*ARBP*	1.46	*18S*	1.99	*ARBP*	1.98	*18S*	2.10
8	*18S*	3.24	*18S*	3.45	*18S*	2.27	*ARBP*	2.41	*α-tubulin*	2.06	*ARBP*	2.17
9	*ARBP*	4.19	*28S*	4.62	*α-tubulin*	2.54	*28S*	2.77	*28S*	2.31	*28S*	2.85
10	*28S*	4.69	*ARBP*	5.55	*28S*	3.33	*α-tubulin*	3.84	*18S*	2.38	*α-tubulin*	3.15

The stability of the reference genes is represented by the coefficient of variation (CV), and the most stable gene has the lowest CV value.

### Stability evaluation using RefFinder

**Figure 5:**
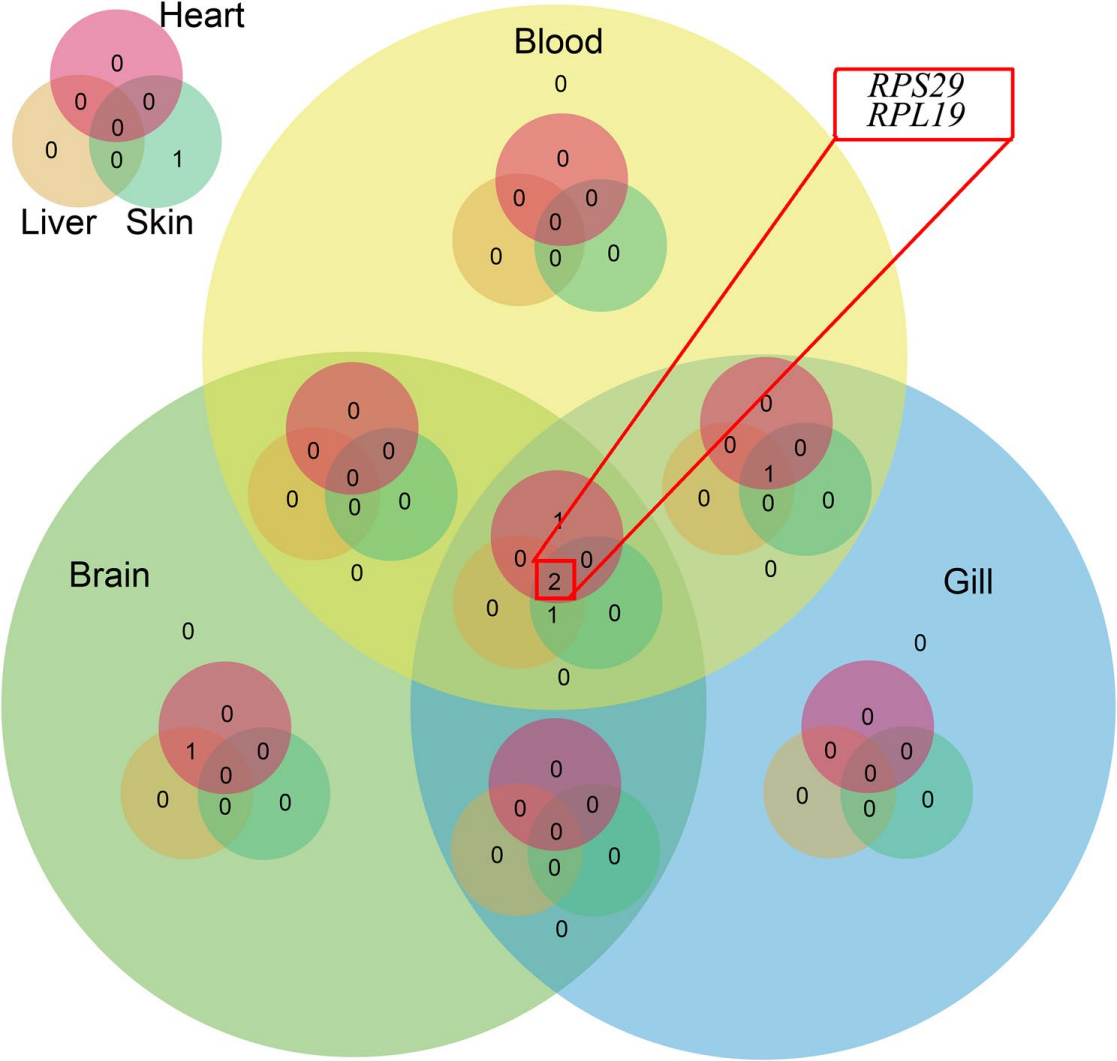
A Venn diagram of the top five candidate taimen reference genes. *RPS29* and *RPL19* (marked with the red rectangle) were stable in all of the tissues, which indicated that they are suitable reference genes for the six tissues, as well as different heat-stress conditions.

Although geNorm, NormFinder, and BestKeeper generated different stability rankings because of their different algorithms, the top five genes were consistent. Furthermore, RefFinder was used to assess the stability of the 10 reference genes, and its results were combined with those of the other three methods to produce the final ranking (Table 4). In the liver and skin under different heat-stress conditions, the most stable reference gene was *RPS29*, followed by *RPL13*. In the gill and blood, the most stable reference genes were *RPL19* and *Saha*, whereas in the heart, the two most stable genes were *RPS29* and *RPL19*. In the brain, *β-actin* and *RPS29* were the most stably expressed genes. A Venn diagram was constructed using the five most stably expressed genes from the six tested tissues, and it indicated that *RPS29* and *RPL19* were the most suitable reference genes for the six tissues, as well as the different heat-stress conditions, followed by *Saha* and *RPL13* (Fig. 5).

**Table 4:** Expression stability values of 10 candidate taimen reference genes under heat-stress conditions as assessed by RefFinder.

Stability ranking	Liver	Brain	Skin	Gill	Heart	Blood
	Gene	Gene	Gene	Gene	Gene	Gene
1	***RPS29***	*β-actin*	***RPS29***	***RPL19***	***RPS29***	*Saha*
2	*RPL13*	***RPS29***	*RPL13*	*Saha*	***RPL19***	***RPL19***
3	*β-actin*	***RPL19***	*Saha*	***RPS29***	*GAPDH*	***RPS29***
4	***RPL19***	*RPL13*	*GAPDH*	*RPL13*	*Saha*	*GAPDH*
5	*GAPDH*	*Saha*	***RPL19***	*GAPDH*	*β-actin*	*RPL13*
6	*18S*	*GAPDH*	*β-actin*	*β-actin*	*ARBP*	*β-actin*
7	*Saha*	*18S*	*18S*	*18S*	*RPL13*	*18S*
8	*α-tubulin*	*α-tubulin*	*ARBP*	*ARBP*	*α-tubulin*	*ARBP*
9	*ARBP*	*28S*	*α-tubulin*	*28S*	*18S*	*28S*
10	*28S*	*ARBP*	*28S*	*α-tubulin*	*28S*	*α-tubulin*

Genes marked with bold were the optimal combination of reference genes in the six tissues.

### Validation of the selected reference genes

**Figure 6:**
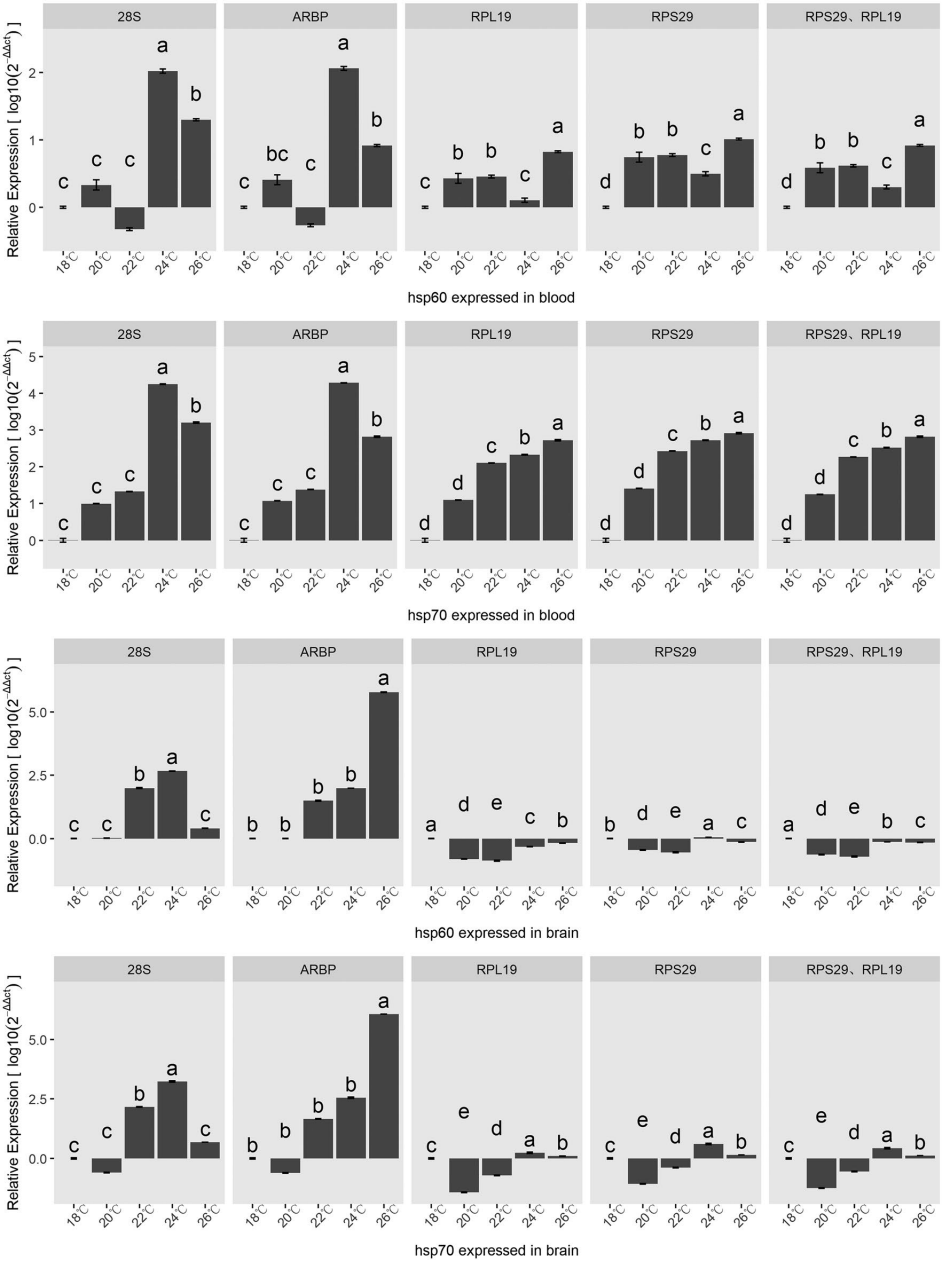
Validation of candidate taimen reference gene stability levels. The expression levels of *hsp60* and *hsp70* in the blood and brain under different heat stress conditions were determined using various candidate references genes. Data represent the means ± SEMs of the log10 values of three biological replicates. Different lowercase letters in the same group indicate significant differences among treatments within each normalization factor (p = 0.05, Tukey’s HSD test).

The qRT-PCR analysis of *hsp60* and *hsp70* showed that the expressed levels of *hsp60* and *hsp70* significantly increased in the blood of taimen under all of the heat-stress conditions if *RPL19*, *RPS29*, and their combination were used as reference genes. However, if *28S rRNA* and *ARBP* were used as reference genes, *hsp60* and *hsp70* were significantly differentially expressed only in the blood of taimen under heat-stress conditions of 24 °C-24 h and 26 °C-24 h (Fig. 6). In the brain tissues, if *RPL19*, *RPS29*, and their combination was used as reference genes, the expression of *hsp60* and *hsp70* in taimen under heat stress showed significant differences compared to that under normal conditions (18 °C-24 h). The expression of *hsp60* and *hsp70* only presented significant differences in taimen under heat-stress conditions of 22 °C-24 h and 24 °C-24 h if 28S was used as reference, and only presented significant differences in taimen under heat-stress conditions of 26 °C-24 h if *ARBP* was used as reference (Fig. 6).

The trends in the significance of the *hsp60* and *hsp70* expression profiles at different temperatures (*p* < 0.05) were consistent when *RPS29* + *RPL19*, *RPS29*, or *RPL19* were used as reference gene(s). In comparison, when *28S rRNA* or *ARBP* was used as the reference gene, the significant differences in the target genes’ expression levels were completely opposite among different groups (*p* < 0.05).

To test whether the reference genes influenced the relative quantification of *hsp60* and *hsp70* in the blood and brain tissues, we performed a two-way ANOVA and found that the reference genes did not influence the relative quantification of *hsp60* and *hsp70* in blood(*P* > 0.05), the variance mainly attributed to samples under different heat stress conditions, the statistical effect sizes of reference genes were 0.06 and 0.03 for *hsp60* and *hsp70*, respectively, and the effect sizes of heat stress conditions were 0.46 and 0.88 for *hsp60* and *hsp70*, respectively. Whereas reference genes significantly affected the relative quantification of *hsp60* and *hsp70* in brain(*P* < 0.01), there were significant differences of relative quantification between *RPL19*, *RPS29*, *RPL19* combined with *RPS29* against *ARBP*, *28S* (*P* < 0.01), the effect sizes of reference genes were same for *hsp60* and *hsp70*, with the value of 0.48, and the effect sizes of heat stress conditions were 0.26 and 0.48 for *hsp60* and *hsp70*, respectively.

## Discussion

Taimen is a high-quality cold freshwater fish. Owing to its water temperature restrictions, it is mainly cultured in southwestern and northern China, but water resources are limited in these regions^42^. Whether it is possible to breed high-temperature-tolerant varieties using gene function and molecular research to expand the culture area for taimen has not been reported. Therefore, this study focused on the importance of water temperature and screened for taimen reference genes suitable for use when fishes were grown under high-temperature conditions. These findings lay a foundation for the study of gene expression during temperature changes and provides a theoretical basis for breeding new taimen varieties with high temperature tolerance.

With the development of functional genomics, gene expression analysis methods are also evolving^43^. Real-time fluorescence quantitative PCR technology is a conventional gene expression analysis method. To eliminate errors caused by technicians or template factors during qRT-PCR, reference genes are often used to normalize the expression levels of target genes^44^. The ideal reference gene should be stably expressed in all tissues and cell types without being affected by internal or external factors^27^. However, owing to differences in species ^45,46^, tissue functions^47,48^, and metabolic characteristics^49,50^, it is almost impossible to obtain universal reference genes.

Many researchers optimize the reference genes used for different species. For example, *RPL13* is the most stable internal reference gene in various tissues of naked schizothoracin (*Oxygymnocypris stewarti*)^51^, whereas *UBCE* and *18S rRNA* are the most stable genes in various tissues of Nile tilapia (*Oreochromis niloticus*)^52^. *RPL17* and *ACTB* have the highest stable expression levels in various tissues of Korean rockfish (*Sebastes schlegelii*)^53^, whereas *B2M* and *18S rRNA* have the highest stable expression levels in pompano (*Trachinotus ovatus*)^54^. In addition, in different tissues of the same species and under varied experimental conditions, the selection of internal reference genes is also different. For example, *sep15* and *metap1* have been selected as stably expressed reference genes in different zebrafish tissues, but *RPL13a* and *RP1P0* are the most stably expressed genes after chemotherapy stimulation^55^. Therefore, it is necessary to characterize the expression of the reference genes under specific experimental conditions.

Four software programs, geNorm, NormFinder, BestKeeper, and RefFinder, are commonly used to assess the stability of reference genes. The geNorm software^27^ may be used to screen reference genes for real-time PCR and to determine the number of optimal reference genes under specific conditions. NormFinder^28^ uses a calculation principle similar to that of geNorm. However, NormFinder not only compares the expression differences of candidate genes, but it also calculates the variation between sample groups. BestKeeper^29^ analyzes the expression values of reference and target genes. RefFinder^30^ evaluates reliable reference genes, including miRNAs, for gene expression analyses by integrating the main calculation programs currently available (geNorm, NormFinder, BestKeeper, and the comparative delta-Ct method). It assigns appropriate weights to individual genes according to the ranking of each program, and then it calculates the geometric means of its weights to form a final ranking. These four software programs have their own characteristics and should be considered comprehensively. In this study, we used a combination of these four programs to identify stably expressed taimen genes in different tissues during temperature changes. geNorm identified *RPS29*, *RPL13* and *RPL19* as being the most stable, whereas 2*8S rRNA*, *ARBPR*, and *β-actin* were the most unstable. This was consistent with the results of BestKeeper. NormFinder identified *RPS29*, *β-actin*, and *Saha* as being more stable in the tissues than *RPL13* and *RPL19*. This differed from the results of geNorm and BestKeeper. The inconsistent results might be due to the co-regulation of the reference genes. The four software were designed dependent on the assumption that the evaluated genes are not co-regulation, in this study, the 10 reference genes were involved in cell skeleton, metabolism, and ribosome, although the public databases show that there is no connection between them, it is unclear whether they are co-regulation. However, all three programs identified *28S rRNA*, *ARBP*, and *18S rRNA* as being unstable. The comprehensive analysis by RefFinder identified *RPS29* and *RPL19* as being stable in all of the tested tissues, followed by *RPL13* and *Saha*, which was almost consistent with the results of the first three analyses.

The stability of reference genes can also be affected by gene duplication. Salmonids underwent the fourth round whole genome duplication event, which caused many genes to replicate followed by pseudogenization, neofunctionalization, and duplication. These paralogous genes might differentially express in tissues and cause difficulty in designing primers to amplify specific copies due to the high similarity between paralogous genes. Among the 10 reference genes selected in this study, the primers of *RPL19*, *RPS29*, and alpha-tubulin could be used to amplify one gene by aligning the primer sequences to the huchen (*Hucho hucho*) genome, two or more homologous genes of the other primers were obtained which might affect the expression stability in tissues. Therefore, gene duplication should be considered to design primers for reference genes, which might hinder the gene expression analysis in non-model species without genomic background information.

To validate the reference genes, we carried out the expression analysis of *hsp60* and *hsp70* in the blood and brain. When the most stable genes were used to normalize the CT values, the expression of the target gene in the blood and brain significantly increased under heat stress and the expression patterns were consistent. When unstable genes (28S and ARBP) were used to normalize the CT values, the expression patterns of the target genes in the blood and brain were different and the expression levels showed fluctuating changes, which were also different from the results normalized by *RPS29*, *RPL19*, and their combination. Although few studies have reported the expression profiles of *hsp60* and *hsp70* in the blood and brain under heat stress, the combination of *RPL19* and *RPS29* might be an optimal reference gene set based on the assumption that the expression profiles should be similar regardless of the reference genes used. These results further demonstrate the importance of selecting the right reference gene for analytical purposes.

As immunomodulators^56^, heat shock proteins have been widely tested as heat-stress indicator proteins in fish. For example, Liu et al.^57^ found that HSP40, HSP70, and HSP90 family members were significantly upregulated in the gill and liver of hybrid catfish under heat stress by RNA-Seq analysis; Smith et al.^58^ showed that *hsp60*, *hsp70*, and *hsp90A* were significantly up-regulated in the liver of crimson spotted rainbow fish (*Melanotaenia duboulayi*) under heat stress; and Shi et al.^59^ found that *hsp60* was significantly up-regulated in the gill, liver, spleen, and head kidney of rainbow trout after more than 4 h at 25 °C, which was similar to the results of the present study. In the present study, the expressed levels of *hsp70* in the blood were significantly increased and presented an upward trend as the water temperature rose, which implied that the expression of *hsp70* in blood might be a potential and convenient marker for heat stress in taimen.

*RPS29*, *RPL13*, and *RPL19* encode large ribosomal subunits, and they are highly conserved in eukaryotes. They are not only involved in protein synthesis, but also in the processes of replication, transcription, RNA processing, DNA repair, self-translational regulation, and developmental regulation. Therefore, these genes can be used as an internal reference genes^47^. The present study showed that the expression levels of *RPS29* and *RPL19* in different taimen tissues under heat-stress conditions were more stable compared with those of other candidate reference genes. Therefore, we recommend *RPS29* and *RPL19* as reference genes for future studies in taimen.

## Supplementary Information

Supplementary Information.
