# Supplementary material for: Evaluation of qPCR reference genes for taimen (Hucho taimen) under heat stress
Source: Sci Rep. 2022 Jan 10;12:313. doi: 10.1038/s41598-021-03872-x (PMC8748915; doi:10.1038/s41598-021-03872-x)
Supplement: Supplementary file 1 — Supplementary Information. [file 41598_2021_3872_MOESM1_ESM.docx]

# Evaluation of qPCR reference genes for taimen (*Hucho taimen*) under heat stress

Xiaoxing Yang^1,2,5, #^; Guangxiang Tong^1,2,3,#^; Le Dong^1,2,5^; Ting Yan^1,2^; Huan Xu^1,2^; Guopan Tang^4^; Yongquan Zhang^1,3^; Kai Ma^1,3^; Jiasheng Yin^1,2,3^; Youyi Kuang^1,2*^

**Affiliation**

1. Heilongjiang River Fisheries Research Institute, Chinese Academy of Fishery Sciences, Harbin 150070, China;

2. Key Laboratory of Freshwater Aquatic Biotechnology and Breeding, Ministry of Agriculture and Rural Affairs, Harbin 150070, China;

3. Key Open Laboratory of Cold Water Fish Germplasm Resources and Breeding of Heilongjiang Province, Harbin 150070, China;

4. Henan University of Animal Husbandry and Economy, Zhengzhou 450016, China;

5. College of Fisheries and Life Science, Shanghai Ocean University, Shanghai 201303, China

# these authors contributed equally to this work.

* To whom correspondence should be addressed. Youyi Kuang, E-mail: kuangyouyi@hrfri.ac.cn.

**Supplementary Figure S1. The gel electrophoresis of the PCR products amplified for 10 reference genes.**

**Supplementary Figure S2. The mean CT values of 10 reference genes in each of the tissues among different experimental conditions.**

**Supplementary Figure S3. The mean CT values of 10 reference genes in each of the experimental conditions among different tissues.**

**Supplementary Table S1. The mean CT values and coefficient of variants of 10 reference genes in each of the tissues among different experimental conditions.**

**Supplementary Table S2. The mean CT values and coefficient of variants of 10 reference in genes each of the experimental conditions among different tissues.**

**Supplementary Table S3. The statistical effect sizes of tissues and heat-stress conditions contributed to CT values of 10 reference genes.**

**Supplementary Figure S1. The gel electrophoresis of the PCR products amplified for 10 reference genes.**


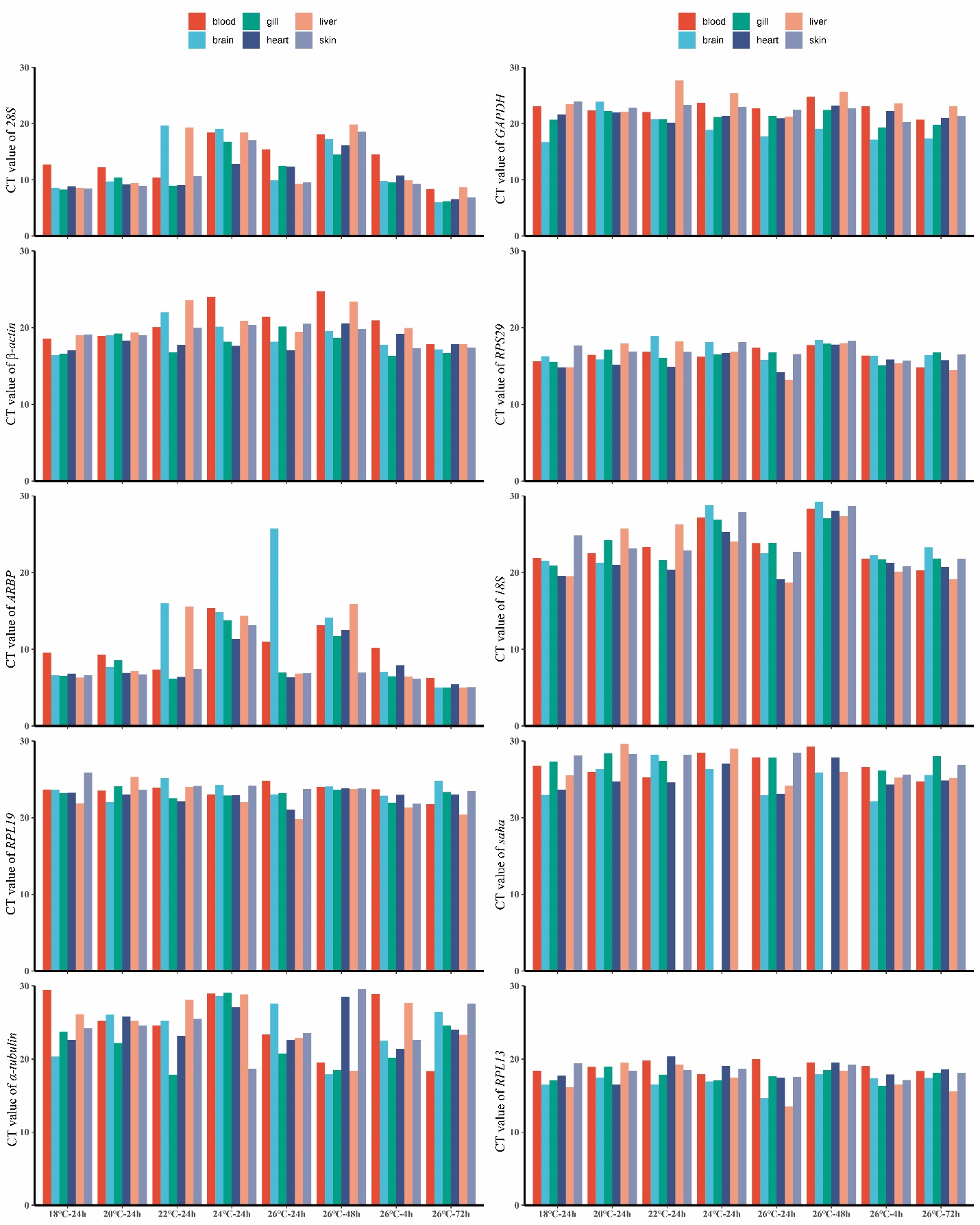


**Supplementary Figure S2. The mean CT values of 10 reference genes in each of the tissues among different experimental conditions.**


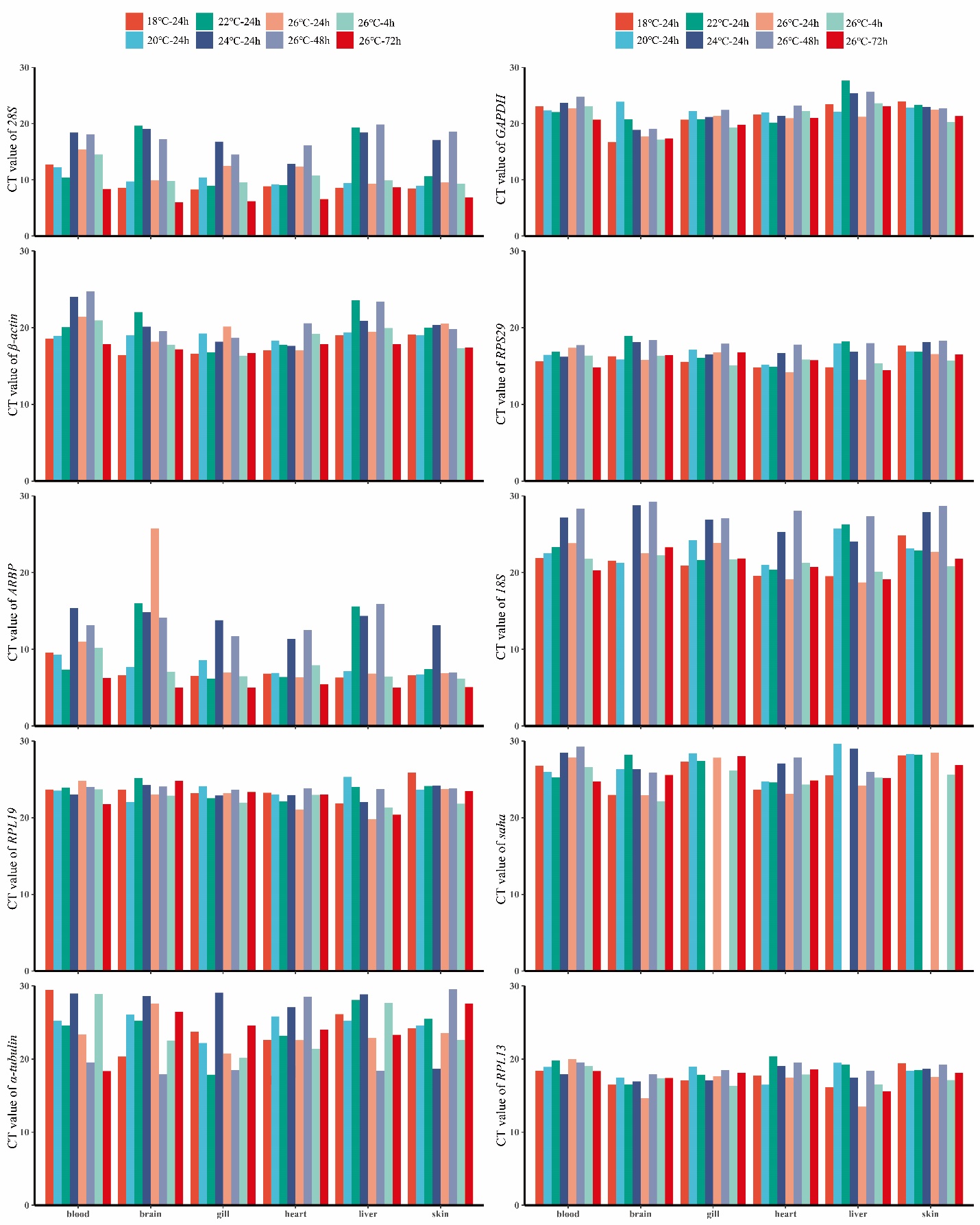


**Supplementary Figure S3. The mean CT values of 10 reference genes in each of the experimental conditions among different tissues.**

**Supplementary Table S1. The mean CT values and coefficient of variants of 10 reference genes in each of the tissues among different experimental conditions.**

| Gene | Tissues | | | | | |
| --- | --- | --- | --- | --- | --- | --- |
|  | Skin | Blood | Heart | Liver | Brain | Gill |
| *28S* | 11.17(4.26%) | 13.77(3.54%) | 10.73(2.99%) | 12.94(5.21%) | 12.49(5.30%) | 10.89(3.49%) |
| *GAPDH* | 22.49(1.15%) | 22.80(1.19%） | 21.59(0.92%) | 24.05(2.09%) | 18.95(2.40%) | 20.99(1.08%) |
| *β-actin* | 19.20(1.25%) | 20.82(2.50%) | 18.18(1.19%) | 20.44(2.07%） | 18.77(1.80%) | 17.82(1.44%) |
| *RPS29* | 17.07(0.88%) | 16.43（0.93%） | 15.64(1.15%) | 16.11(1.90%) | 17.01(1.24%) | 16.48(0.89%) |
| *ARBP* | 7.38(2.43%) | 10.28(2.94%) | 7.96(2.56%) | 9.71(4.69%) | 12.14(6.97%) | 8.17(3.06%) |
| *18S* | 24.11(2.82%) | 23.66(2.77%) | 21.94(3.11%) | 22.63(3.6%) | 24.94(3.89%) | 23.53(2.42%) |
| *RPL19* | 23.82(1.10%) | 23.56(0.88%) | 22.78(0.83%) | 22.31(1.9%) | 23.74(1.04%) | 23.10(0.66%) |
| *saha* | 28.29(1.61%) | 26.86(1.56%) | 25.02(1.64%) | 27.02(2.65%) | 25.04(2.11%) | 28.18(1.41%) |
| *α-tubulin* | 26.13(3.09%) | 28.59(4.13%) | 24.40(2.48%) | 26.76(3%） | 25.87(3.18%) | 25.38(4.56%) |
| *RPL13* | 18.37(0.77%) | 19(0.75%) | 18.39(1.25%) | 17.04(2.02%) | 16.84(1.02%) | 17.69(0.85%) |

**Supplementary Table S2. The mean CT values and coefficient of variants of 10 reference genes in each of the experimental conditions among different tissues.**

| Gene | Heat stressed condition | | | | | | | |
| --- | --- | --- | --- | --- | --- | --- | --- | --- |
|  | 18℃-24h | 20℃-24h | 22℃-24h | 24℃-24h | 26℃-4h | 26℃-24h | 26℃-48h | 26℃-72h |
| *28S* | 9.26(1.71%) | 9.99(1.22%) | 12.99(5.07%) | 17.10(2.27%) | 10.63(1.97%) | 11.51(2.38%) | 17.41(1.9%) | 7.11(1.14%) |
| *GAPDH* | 21.6(2.69%) | 22.58(0.71%) | 22.47(2.78%) | 22.26(2.26%) | 20.96(2.47%) | 21.08(1.78%) | 23(2.28%) | 20.55(1.93%) |
| *β-actin* | 17.78(1.26%) | 18.98(0.36%) | 20.04(2.54%) | 20.20(2.28%) | 18.58(1.75%) | 19.46(1.61%) | 21.12(2.38%) | 17.48(0.48%) |
| *RPS29* | 15.79(1.08%) | 16.58(1%) | 16.98(1.44%) | 17.08(0.81%) | 15.78(0.51%) | 15.65(1.61%) | 18.02(0.27%) | 15.78(0.96%) |
| *ARBP* | 7.08(1.22%) | 7.74(1.01%) | 9.83(4.66%) | 13.81(1.44%) | 7.39(1.52%) | 10.65(7.61%) | 12.41(3.03%) | 5.3(0.5%) |
| *18S* | 21.38(1.96%) | 22.98(1.79%) | 24.19(3.69%) | 26.71(1.73%) | 21.34(0.75%) | 21.81(2.29%) | 28.13(0.82%) | 21.2(1.45%) |
| *RPL19* | 23.58(1.3%) | 23.60(1.08%) | 23.66(1.12%) | 23.21(0.84%) | 22.43(0.89%) | 22.61(1.85%) | 23.85(0.16%) | 22.79(1.52%) |
| *saha* | 25.73(2.05%) | 27.21(1.85%) | 27.53(2.48%) | 28.51(1.57%) | 25.01(1.61%) | 25.71(2.60%) | 28.31(2.08%) | 25.87(1.31%) |
| *α-tubulin* | 24.42(3.12%) | 24.87(1.41%) | 26.50(3.31%) | 28.99(1.37%) | 23.89(3.55%) | 23.46(2.26%) | 30.88(2.41%) | 26.48(3.54%) |
| *RPL13* | 17.54(1.23%) | 18.28(1.11%) | 18.73(1.40%) | 17.86(0.85%) | 17.37(0.99%) | 16.79(2.35%) | 18.84(0.68%) | 17.70(1.10%) |

**Supplementary S3. The statistical effect sizes of tissues and heat-stress conditions contributed to CT values of 10 reference genes.** The effect size was represented by partial Eta squared calculated with the EtaSq function in the DescTools R package.

| Gene | Effect sizes | |
| --- | --- | --- |
|  | Tissues | Heat stress conditions |
| *18S* | 0.27 | 0.71 |
| *28S* | 0.25 | 0.75 |
| *α-tubulin* | 0.22 | 0.50 |
| *ARBP* | 0.25 | 0.49 |
| *β-actin* | 0.46 | 0.49 |
| *GAPDH* | 0.63 | 0.32 |
| *RPL13* | 0.41 | 0.34 |
| *RPL19* | 0.25 | 0.22 |
| *RPS29* | 0.26 | 0.48 |
| *saha* | 0.52 | 0.49 |
